# Low-Dose Sirolimus Immunoregulation Therapy in Patients with Active Rheumatoid Arthritis: A 24-Week Follow-Up of the Randomized, Open-Label, Parallel-Controlled Trial

**DOI:** 10.1155/2019/7684352

**Published:** 2019-11-03

**Authors:** Hong-Yan Wen, Jia Wang, Sheng-Xiao Zhang, Jing Luo, Xiang-Cong Zhao, Chen Zhang, Cai-Hong Wang, Fang-Yuan Hu, Xiao-Juan Zheng, Ting Cheng, Hong-Qing Niu, Guang-Ying Liu, Wen-Xian Yang, Na-Na Yu, Jin-Li Ru, Qi-Xiang Chen, Xue-Chun Lu, Pei-Feng He, Chong Gao, Xiao-Feng Li

**Affiliations:** ^1^Department of Rheumatology, The Second Hospital of Shanxi Medical University, Taiyuan, Shanxi, China; ^2^NCPC New Preparation Branch Factory, North China Pharmaceutical Co., Ltd., 115 Hainan Road, Shijiazhuang, Hebei, China; ^3^Department of Hematology, PLA General Hospital, Beijing, China; ^4^Department of Medical Information Management, Shanxi Medical University, Taiyuan, Shanxi, China; ^5^Department of Pathology, Brigham and Women's Hospital, Harvard Medical School, Boston, MA, USA

## Abstract

**Background:**

We have reported previously the insufficient absolute number or functional defects of regulatory T cells (Tregs) in patients with rheumatoid arthritis (RA), challenging conventional unspecific immunosuppressive therapy. Sirolimus, a mTOR inhibitor, is reported to allow growth of functional Tregs; here, we investigated the efficacy of low-dose sirolimus combined with conventional immunosuppressants (sirolimus immunoregulation therapy) for RA treatment with lower side effects and better tolerance.

**Methods:**

In this nonblinded and parallel-group trial, we randomly assigned 62 patients to receive conventional glucocorticoids and immunosuppressants with or without sirolimus at a dosage of 0.5 mg on alternate days for 24 weeks in a 2 : 1 ratio. The demographic features, clinical manifestations, and laboratory indicators including peripheral blood lymphocyte subgroups and CD4^+^T subsets were compared before and after the treatment.

**Results:**

Finally, 37 patients in the sirolimus group and 18 in the conventional treated group completed the 6-month study. By 24 weeks, the patients with sirolimus experienced significant reduction in disease activity indicators including DAS28, ESR, and the number of tender joints and swollen joints (*p* < 0.001). Notably, they had a higher level of Tregs as compared with those with conventional therapy alone (*p* < 0.05), indicating that sirolimus could partly restore the reduced Tregs. Concomitantly, their usage of immunosuppressants for controlling disease activity was decreased as compared with the conventional group with no difference in blood routine, and liver and renal functions both before and after the treatment of sirolimus and between the two groups (*p* > 0.05).

**Conclusions:**

Low-dose sirolimus immunoregulatory therapy selectively upregulated Tregs and partly replaced the usage of immunosuppressants to control disease activity without overtreatment and evaluable side effect. Further study is required using a large sample of RA patients treated with sirolimus for a longer period. This trial is registered at the Chinese Clinical Trial Registry (http://www.chictr.org.cn/showproj.aspx?proj=17245).

## 1. Introduction

Rheumatoid arthritis (RA) is a chronic autoimmune disease, potentially leading to joint cartilage and bone damage and even disability due to profound inflammation [1]. Nonsteroidal anti-inflammatory drugs (NSAIDs), corticosteroids, and immunosuppressants are conventionally used to treat RA patients [1]. However, a portion of patients still have inadequate response to them or severe side effects. New therapies are urgently required for RA.

Many important immunological dimensions, especially the balance of effector T cells and regulatory T cells (Tregs), are altered in RA [2, 3]. Th17 cells, one of the effector T subsets among CD4^+^T cells, have been reported to mediate the inflammatory process by producing interleukin 17 (IL-17) as well as other effector cytokines and chemokines [4–6]. In contrast, Tregs actively suppress activation of the immune system and prevent autoimmune disease [7]. Recently, we have reported the reduction of the absolute number of peripheral Tregs but not the increase of effector T and Th17 cells in RA patients [8, 9], which argues and challenges the pathological foundation of conventional immunosuppressive therapy that also nonspecifically inhibits Tregs. Thus, one of goals of a new therapy for RA should be to maintain and restore a relative balance between effector T and Treg cells.

Sirolimus, also known as rapamycin, is a macrolide compound that inhibits its mechanistic target (mTOR), which regulates cell growth and metabolism in response to environmental cues. mTOR is also essential in driving abnormal lineage specification within the immune system in various rheumatic diseases [10]. Several studies have reported that sirolimus and its analogues reduced joint inflammation in animal models of arthritis [11] and in a few patients with RA [12–14] or JIA [15]. Further study revealed that this clinical benefit might accrue from the inhibition of mTOR activation in the growth of synovial fibroblast cells [16].

However, the clinical application of sirolimus therapy in RA patients is few up to now, and the evaluation of therapeutic efficiency of mTOR inhibitors in active RA is very limited. Furthermore, a relatively higher dose of sirolimus that was used before has side effects [17]. In this study, we initiated firstly this prospective study to assess safety, tolerance, efficacy, and the status of immunological cells in patients with active RA treated with low-dose sirolimus combined with original therapy.

## 2. Methods

### 2.1. Study Design and Participants

To assess the efficacy and safety of sirolimus for patients with RA, we did a prospective, single-arm, open-label, phase 1/2 trial at the Department of Rheumatology, Second Hospital of Shanxi Medical University (Taiyuan, China), with approval from the Second Hospital of Shanxi Medical University Ethics Committee (ethics number: 2016-KY-014). This trial is registered at the Chinese Clinical Trial Registry (number ChiCTR-IPR-17010307).

All the patients fulfilled the 1987 and 2010 rheumatoid arthritis classification criteria [18, 19]. These patients enrolled in the study were aged between 18 and 65 years and had active disease (DAS28-ESR scores > 3.2). The patients were excluded from this study if they were allergic or intolerant to sirolimus, suffering malignant disease, had a history of malignancy, or had a recent clinically significant infection.

### 2.2. Procedures

The patients had a complete physical examination before enrolment and were randomly assigned (2 : 1) to the sirolimus group and the conventional group. All patients were freely receiving prednisone and other immunosuppressive medications to control disease activity to meet the treat-to-target (T2T) recommendations [20]. Patients in the sirolimus group received additional oral sirolimus (ordered from North China Pharmaceutical Co. Ltd.) at a dosage of 0.5 mg per other day.

Patients were treated with sirolimus for 6 months. The clinical and laboratory indicators were assessed on week 0 (before administration of the first sirolimus dose), and week 3, week 6, week 12, and week 24 after initiation of sirolimus treatment. Laboratory tests included complete blood counts, erythrocyte sedimentation rate (ESR), liver and kidney function tests, and urinalysis. Assessments of flow cytometry for peripheral blood lymphocyte subgroups and CD4^+^T subsets are described in the supplementary materials (supplementary [Supplementary-material supplementary-material-1]). Treatment was discontinued if the patients developed infections, which could not be controlled within 5 days after intravenous antibiotics therapy.

### 2.3. Outcomes

The primary efficacy endpoints were a decrease in disease activity, defined as a decrease in DAS28-ESR scores at each visit during treatment compared with the baseline. Secondary endpoints were a decrease in doses of prednisone or disease-modifying antirheumatic drugs (DMARDs) required to control disease activity and changes in immunobiological biomarkers of clinical responsiveness compared to conventional groups.

Safety outcomes included tolerance as assessed by the occurrence of common side effects. The development of nonhealing oral ulcers or a new onset headache indicated intolerance to sirolimus. Thrombocytopenia, mucositis, oedema, and proteinuria, which have been observed in renal transplant patients, were also monitored as safety outcomes.

### 2.4. Statistical Analysis

The demographic parameters of the control and sirolimus-treated patients were compared using an unpaired *t*-test for parametric data (age, PB lymphocyte subpopulations, and CD4^+^T subsets as well as blood routine, and liver and renal function) and the *χ*^2^ test for proportions (sex) and drug usage. Repeated measure mixed model logistic regression analysis was used to assess the effects of treatments on clinical indices and biomarkers recorded at weeks 3-24 compared with week 0. All *p* values reported herein are two-tailed. *p* value < 0.05 was taken as statistical significance. The software for the statistics was SPSS 22.0 and GraphPad Prism6.0.

## 3. Results

**Figure 1:**
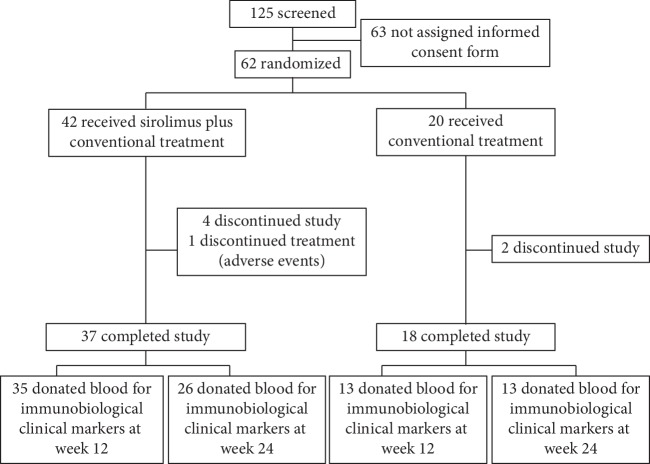
Disposition of patients in the trial.

Between April 7, 2017, and February 9, 2018, 62 patients signed the informed consent form and were enrolled in this study. One of the consented patients was excluded for not meeting eligibility criteria after screening (patient number: 025, DAS28 < 3.2). Only one of them discontinued sirolimus treatment because of intolerance (patient number: 028).The mean age was 50.3 ± 10.6 years in the sirolimus group and 51.8 ± 8.7 years in the conventional group (*t* = 0.563, *p* > 0.05), and 44 (77.2%) patients were female, and there was no difference in proportions of sex between the two groups (*χ*^2^ = 0.152, *p* > 0.05). Baseline clinical characteristics of all enrolled patients, including age, sex, disease duration, DAS28-ESR index score, prednisone dose, and usage of immunosuppressant medication, are shown in the Table 1 and supplementary [Supplementary-material supplementary-material-1]. Finally, 55 (88.7%) patients completed 6 months of treatment (sirolimus group *n* = 37; conventional group *n* = 18). 48 (87.3%) of 55 eligible patients donated their blood and tested their immunobiological biomarkers at week 12, and 39 (70.9%) of them tested at week 24 after the treatment (Figure 1).

### 3.1. Clinical Efficacy Outcomes

**Figure 2:**
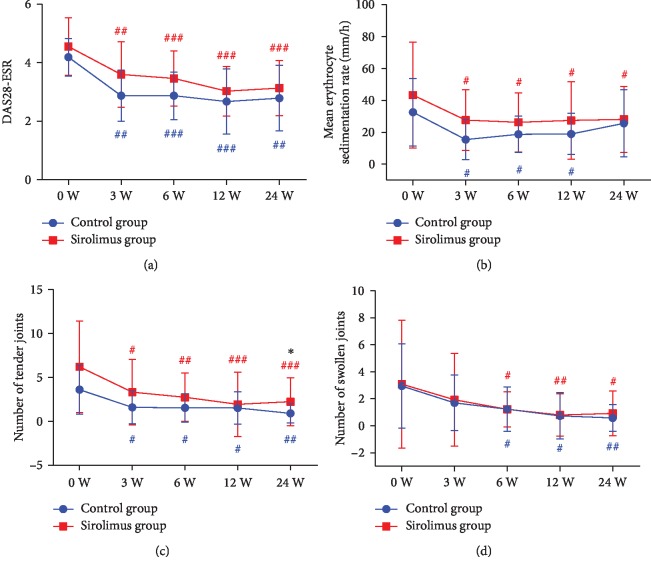
Efficacy of sirolimus in reducing disease activity. Mean DAS28-ESR score (a), ESR (b), mean number of tender joints (c), and swollen joints (d). Overall changes during treatment were assessed by repeated measure analysis using a mixed-effect model. A two-tailed unpaired *t*-test was used to compare the disease activity measures between sirolimus and conventional groups. Error bars show SD. DAS28: 28-joint disease activity score; ESR: erythrocyte sedimentation rate. ^#^*p* < 0.05, ^##^*p* < 0.01, and ^###^*p* < 0.001 relative to baseline (week 0) in the conventional group (blue); ^#^*p* < 0.05, ^##^*p* < 0.01, and ^###^*p* < 0.001 relative to baseline in the sirolimus group (red); ^∗^*p* < 0.05 compared between groups.

In the sirolimus group, the mean DAS28-ESR score decreased from 4.55 ± 0.98 at week 3 to 3.13 ± 0.94 at week 24 (*Z* = −5.130, *p* < 0.001; Figure 2(a)). Other diseases activity measures such as ESR, TJC, and SJC were all significantly reduced during 6 months of treatment with sirolimus (*p* < 0.05; Figure 2(b)–2(d)). There was also a significant decrease of disease activity in the conventional group with a lower level of TJC at 24 weeks; other disease activity indexes were comparable to that of the sirolimus group.

**Figure 3:**
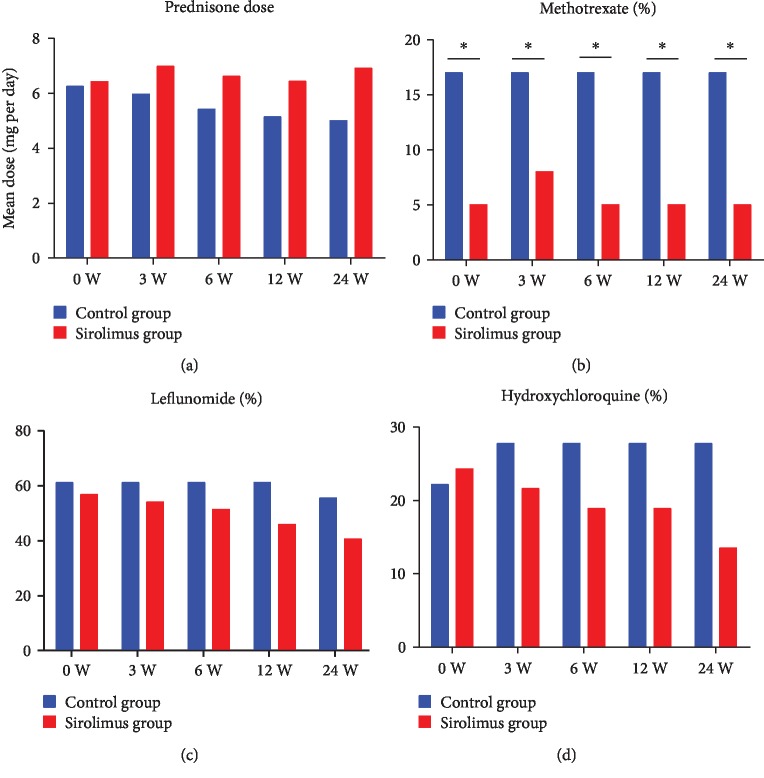
Reductions of drugs required to control disease activity. Mean daily prednisone dose (a), percentage of patients receiving treatment of methotrexate (b), leflunomide (c), and hydroxychloroquine (d) at baseline (week 0) and during treatment (weeks 3, 6, 12, and 24).

To control disease activity to meet the treat-to-target (T2T) recommendations, all patients were free to increase or decrease prednisone or DMARDs. When analyzing details in medication uses, no difference of the mean daily prednisone dose required to control disease activity was observed between the sirolimus and conventional groups (*p* > 0.05; Figure 3(a)). But compared with the conventional group, patients in the sirolimus group had a lower usage rate of DMARDs such as methotrexate, leflunomide, or hydroxychloroquine, which was more observable during the follow-up period (Figure 3(b)–3(d)).

### 3.2. Changes in Immunobiological Biomarkers

**Figure 4:**
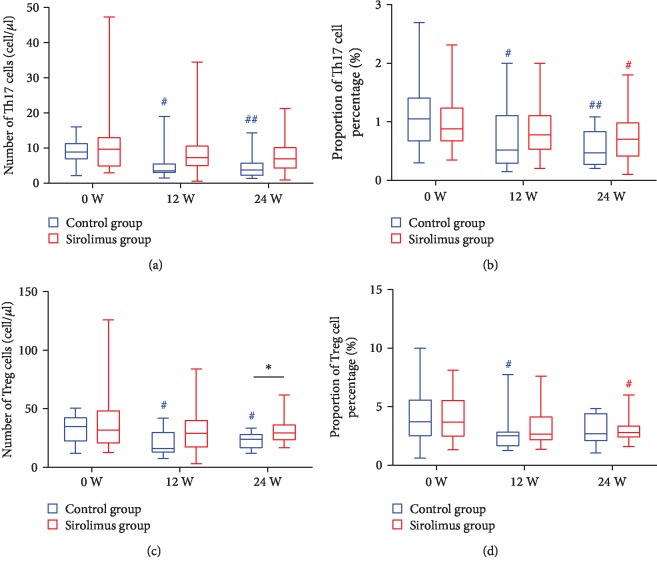
Changes of levels of Th17 (a, b) and Tregs (c, d) after different treatments. Effects of treatments were assessed by repeated measure analysis using a mixed-effect model. (a) and (c) represent the changes in absolute count (median, range) of Th17 and Treg cells, respectively, while (b) and (d) represent the changes in their percentages (median, range). A two-tailed unpaired *t*-test was used to compare the disease activity measures between the sirolimus and conventional groups. ^#^*p* < 0.05 and ^##^*p* < 0.01 relative to baseline (week 0) in the conventional group (blue); ^#^*p* < 0.05 relative to baseline in the sirolimus group (red); ^∗^*p* < 0.05 compared between groups.

Patients treated with conventional immunosuppressants alone had a significant decrease of proinflammatory Th17 cells at week 12 (*Z* = −2.722, *p* < 0.05) and 24 (*Z* = −2.762, *p* < 0.01; Figures 4(a) and 4(b)), but meantime, anti-inflammatory Tregs were also significantly reduced from 32.2 ± 12.1/*μ*l at week 0 to 21.2 ± 11.2/*μ*l at week 12 (*Z* = −2.102, *p* < 0.05) and 23.1 ± 6.4/*μ*l at week 24 (*Z* = −1.882, *p* < 0.05; Figure 4(c)). In contrast, patients who received sirolimus combination treatments had a higher level (31.0 ± 2.1/*μ*l) of Tregs compared with patients (23.1 ± 1.8/*μ*l) who received immunosuppressive therapy alone at week 24 (*Z* = −2.235, *p* < 0.05; Figure 4(c)), indicating that low-dose sirolimus partly reversed the reduction of these cells.

We also assessed the mean proportions and absolute numbers of specific lymphocyte subsets, Th1 and Th2 cells during 24 weeks of treatment. The levels of peripheral blood lymphocyte subgroups and Th2 cells had no significant change, while patients with sirolimus treatment had higher proportion of Th1 cells than did the matched control group at week 12 (supplementary [Supplementary-material supplementary-material-1] and [Supplementary-material supplementary-material-1]).

### 3.3. Safety Outcomes

**Figure 5:**
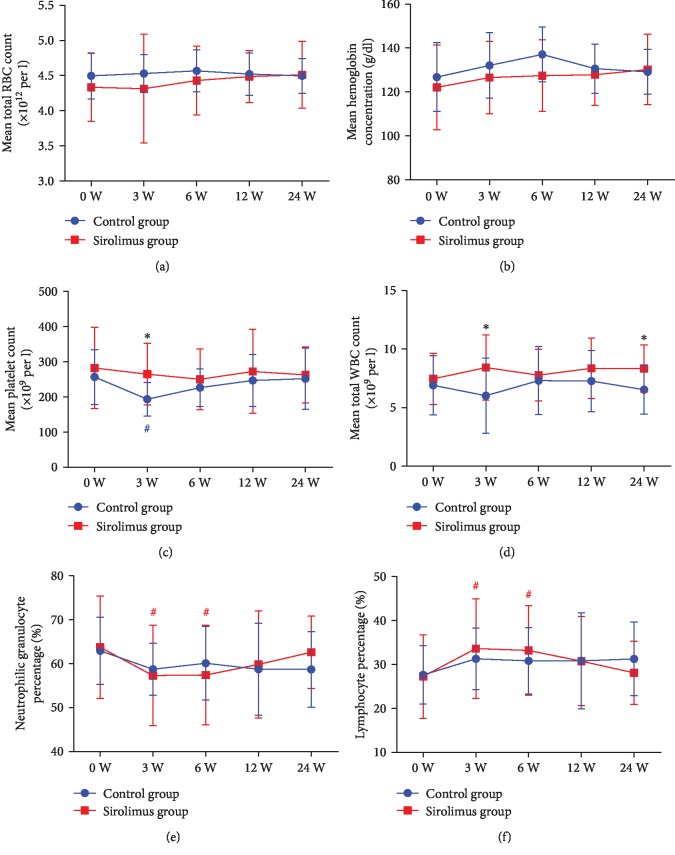
Safety outcomes of blood routine. Mean RBC counts (a), hemoglobin concentration (b), platelet counts (c), total WBC counts (d), and proportion of neutrophils (e) and lymphocytes (f) were measured before treatment (week 0) and after initiation of treatment at weeks 3, 6, 12, and 24 in the control and sirolimus groups. An unpaired *t*-test was used to compare the differences in blood routine measures between the sirolimus and conventional groups. Error bars show SD. RBC: red blood cell; WBC: white blood cell. ^#^*p* < 0.05 compared to the baseline (week 0) in the conventional group (blue); ^#^*p* < 0.05 compared to the baseline in the sirolimus group (red); ^∗^*p* < 0.05 compared between groups.

**Figure 6:**
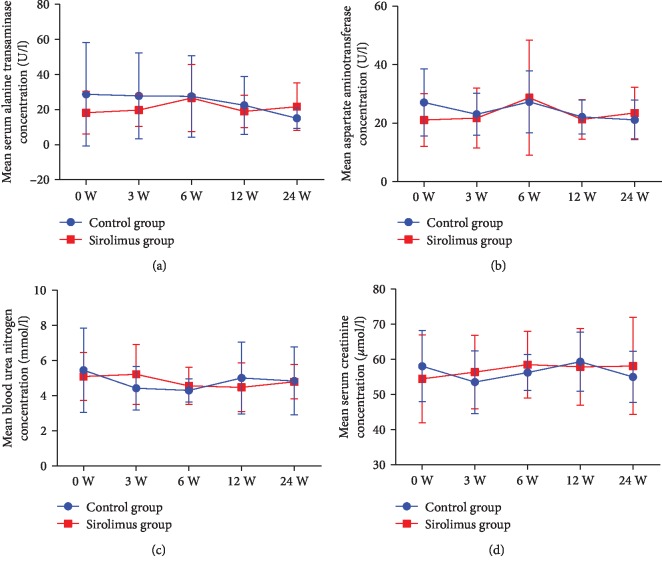
Safety outcomes of liver and renal function. Mean concentration of serum alanine transaminase (a), aspartate aminotransferase (b), blood urea nitrogen (c), and serum creatinine (d) are shown. Overall changes in safety endpoints during treatment were assessed to indicate for each safety outcome. An unpaired *t*-test was used to compare the blood routine measures between the sirolimus and conventional groups. Error bars show SD.

We measured the blood concentration of sirolimus at last visit and found that the concentration was 1.8 ± 0.4 ng/ml, which indeed was much lower than the reported therapeutic range of 6-15 ng/ml [17]. At this low dosage, blood routine tests showed no significant changes in RBC counts (Figure 5(a)) and hemoglobin concentration (Figure 5(b)) compared with the two groups at each time point (*p* > 0.05). Platelet counts in the conventional group were transiently decreased at week 3 compared with the baseline (*Z* = −2.265, *p* < 0.05), which was lower than that of the sirolimus group (*Z* = −2.668, *p* < 0.05), but no statistically significant differences were observed at weeks 6, 12, and 24 between the two groups (Figure 5(c)). Patients treated with conventional immunosuppressants had a decreased tendency of WBC counts (Figure 5(d)), which was slightly lower than that of sirolimus treatments at week 3 (*Z* = −2.360, *p* < 0.05) and week 24 (*Z* = −2.498, *p* < 0.05). Compared with the baseline, sirolimus-treated patients had a lower level of neutrophilic granulocyte percentage at week 3 (*Z* = −1.092, *p* < 0.05) and week 12 (*Z* = −1.091, *p* < 0.05; Figure 5(e)). At the same time, mixed model logistic regression analysis revealed a significant increase in the lymphocyte proportions in the sirolimus group at week 3 (*Z* = −2.037, *p* < 0.01) and week 6 (*Z* = −2.172, *p* < 0.05; Figure 5(f)). Liver function, assessed by aspartate aminotransferase and alanine aminotransferase concentrations, was not affected (*p* > 0.05; Figures 6(a) and 6(b)). Renal function, valued by blood urea nitrogen and serum creatinine, was not affected too (*p* > 0.05; Figures 6(c) and 6(d)). Except for one patient who developed limb oedema after one week of treatment with sirolimus and therefore withdrew from the study, no thrombocytopenia, mucositis, or proteinuria was observed.

## 4. Discussion

mTOR serves as a regulator of growth, proliferation, and survival in eukaryotic cells. This pathway has been well known to play important roles in regulating adaptive and innate immune cell function [21] and is also critical for inflammatory bone destruction in RA [22]. Sirolimus, an mTOR inhibitor, exhibits immunosuppressive effects via inhibition of B cell and T cell proliferation. Therefore, it was initially developed as an immunosuppressant in solid organ transplant setting and as a growth suppressor in the treatment of tumors [23]. Subsequently, the potency of sirolimus in blocking T-cell activation was first found to be beneficial in the treatment of rheumatic diseases in the context of systemic lupus erythematosus (SLE), both in animal models [24] and patients [17, 25]. However, so far, clinical evidence for the use of mTOR inhibitors in RA is very limited. Only one clinical study has been published that showed a moderate effect on the signs and symptoms of disease over 12 weeks of treatment with an mTOR inhibitor in combination with methotrexate [12]. On the other hand, no other study has shown that the absolute number of peripheral Tregs is decreased in RA patients and that mTOR inhibitors affect the levels of Treg and other T cell subsets in RA patients.

Since the main adverse events of sirolimus are dose-dependent [26], our parallel-controlled study provides preliminary evidence that low-dose sirolimus (0.5 mg per other day) combined with conventional immunosuppressive drugs is safe, better tolerated, and clinically efficacious in patients with RA. Lai et al. [17] reported that active patients with systemic lupus erythematosus treated with sirolimus at a starting dosage of 2 mg daily (8 times higher than ours) had reduced hemoglobin and neutrophil counts or extensive oral ulcers. In contrast, except for one patient who suffered from oedema, our low-dose sirolimus therapy did not show evaluable side effects such as cytopenia or ulcer.

Sirolimus has been proven to have antirheumatic properties at the dosage of 2-6 mg once daily [17, 27], with the serum trough level maintained between 4.9 and 15 ng/ml [15, 27]. Our study found that low-dose sirolimus (0.5 mg per other day with the serum concentration of 1.8 ± 0.4 ng/ml) had an immunoregulatory property besides immunosuppression by the rebalancing of Th17 and Tregs. Conventional immunosuppressant medications can alleviate disease activity by inhibiting lymphocytes nonspecifically [28, 29]. Our results showed that conventional immunosuppressive strategy not only decreased the proinflammatory Th17 cells but also reduced anti-inflammatory Tregs, which may aggravate the disturbance of the immune balance. On the contrary, patients treated with sirolimus had higher levels of Tregs compared with those who received conventional treatments, which means low-dose sirolimus could reverse the reduction of Tregs not only by the disease itself but also by immunosuppressive agents. Similarly, Li et al. [30] reported that sirolimus promotes the expression of FoxP3^+^ in CD4^+^T cell subsets and the proliferation of Tregs by inducing TGF-*β* secretion. Biswas et al. [31] found that mTOR inhibitors synergistically promote induction of antigen-specific Tregs via selective expansion of plasmacytoid dendritic cells. These evidences support that sirolimus participates in immunoregulation by augmenting Tregs. Our results indicate that sirolimus can be used to rebalance Th17 and Tregs as an immunoregulatory drug besides an immunosuppressant to treat RA patients. In this study, we defined low-dose sirolimus combined with immunosuppressants to treat RA as sirolimus immunoregulatory therapy.

Importantly, disease activity was significantly reduced by low-dose sirolimus immunoregulatory therapy. Though no differences of disease activity measures other than TJC at week 24 were observed in sirolimus-treated patients compared with those receiving conventional medications, the restoration of Tregs by sirolimus should have longer-term benefit for the remission of the disease and the withdrawal of conventional immunosuppressants. Interestingly, application of immunosuppressants required to control disease activity in sirolimus treatment patients was significantly reduced compared with that in the conventional group, indicating that sirolimus could effectively replace the usage of conventional immunosuppressants.

Overall, this study was the first clinical trial on the effect of low-dose sirolimus on active RA. The sirolimus immunoregulatory therapy not only effectively reverses the reduced Tregs and inhibits effector T cells but also alleviates clinical symptoms and decreases the immunosuppressive applications in patients with active RA, which could avoid overtreatment and evaluable side effects of conventional therapy. More high quality trials with large samples and longer following-up are proposed to clarify the further benefits of sirolimus combination therapies.

## Figures and Tables

**Table 1:** Baseline characteristics of all enrolled patients.

	Sirolimus group	Conventional group
*n*	42	20
Sex (female/male)	34/8	17/3
Age (years), $\bar{x}\pm s$	50.3 ± 10.6	51.8 ± 8.7
Duration of disease (years), median (range)	5 (1-20)	6 (2-14)
DAS28, $\bar{x}\pm s$	4.5 ± 1.1	4.1 ± 0.6
TJC, $\bar{x}\pm s$	6.7 ± 5.9	4.0 ± 2.7
SJC, $\bar{x}\pm s$	3.0 ± 4.5	3.0 ± 3.0
ESR (mm/h), $\bar{x}\pm s$	43.3 ± 33.3	31.0 ± 20.4
Prednisone dose (mg/d), $\bar{x}\pm s$	6.2 ± 5.4	6.2 ± 5.7
Use of concomitant agents (no. of patients)		
Methotrexate	3	5
Leflunomide	23	11
Hydroxychloroquine	10	4
Thalidomide	1	1

## Data Availability

All data generated or analyzed during this study are included in this published article.

## Supplementary Materials

Supplementary Materials Supplementary Table 1: clinical details of patients enrolled in the study. Supplementary Figure 1: phenotypic characterization of lymphocyte subpopulations by flow cytometry. (A) Representative flow cytometry analysis of peripheral lymphocytes. T: CD45^+^CD3^+^; B: CD45^+^CD3^−^CD19^+^; NK: CD45^+^CD3^−^CD16^+^CD56^+^ NK; CD4^+^T: CD45^+^CD3^+^CD4^+^; CD8^+^T: CD45^+^CD3^+^CD8^+^. (B) Representative flow cytometry analysis of CD4^+^T cell subsets. All dot plot analyses are of CD4^+^ gated lymphocyte. Th1: CD4^+^INF-*γ*^+^; Th2: CD4^+^IL-4^+^; Th17: CD4^+^IL-17^+^; Treg: CD4^+^CD25^+^Foxp3^+^. Supplementary Figure 2: comparison of lymphocyte subpopulations between the groups at visits 1, 4, and 5. (A) and (B) represent the T cell level, (C) and (D) the B cell level, (E) and (F) the CD4^+^T cell level, (G) and (H) the CD8^+^T cell level, and (K) and (L) the total lymphocyte cell level. Effects of treatments were assessed by repeated measure analysis using a mixed-effect model. Two-tailed unpaired *t*-test was used to compare the disease activity measures between sirolimus and conventional groups. No significant differences were observed at each visit and between the groups. Supplementary Figure 3: comparison of CD4^+^T cell subsets between the groups before treatment and weeks 6, 12, or 24 after the treatment. (A) and (B) represent the absolute number and percentage of the Th1 cells and (C) and (D) represent those of the Th2 cell levels, respectively. Two-tailed unpaired *t*-test was used to compare the disease activity measures between the sirolimus and conventional groups. ^#^*p* < 0.05 relative to the baseline (week 0) in the sirolimus group (red); ^∗^*p* < 0.05 compared between the groups.
