# Supplementary material for: Low-Dose Sirolimus Immunoregulation Therapy in Patients with Active Rheumatoid Arthritis: A 24-Week Follow-Up of the Randomized, Open-Label, Parallel-Controlled Trial
Source: J Immunol Res. 2019 Nov 3;2019:7684352. doi: 10.1155/2019/7684352 (PMC6874993; doi:10.1155/2019/7684352)
Supplement: Supplementary Materials — Supplementary Table 1: clinical details of patients enrolled in the study. Supplementary Figure 1: phenotypic characterization of lymphocyte subpopulations by flow cytometry. (A) Representative flow cytometry analysis of peripheral lymphocytes. T: CD45+CD3+; B: CD45+CD3−CD19+; NK: CD45+CD3−CD16+CD56+ NK; CD4+T: CD45+CD3+CD4+; CD8+T: CD45+CD3+CD8+. (B) Representative flow cytometry analysis of CD4+T cell subsets. All dot plot analyses are of CD4+ gated lymphocyte. Th1: CD4+INF-γ+; Th2: CD4+IL-4+; Th17: CD4+IL-17+; Treg: CD4+CD25+Foxp3+. Supplementary Figure 2: comparison of lymphocyte subpopulations between the groups at visits 1, 4, and 5. (A) and (B) represent the T cell level, (C) and (D) the B cell level, (E) and (F) the CD4+T cell level, (G) and (H) the CD8+T cell level, and (K) and (L) the total lymphocyte cell level. Effects of treatments were assessed by repeated measure analysis using a mixed-effect model. Two-tailed unpaired t-test was used to compare the disease activity measures between sirolimus and conventional groups. No significant differences were observed at each visit and between the groups. Supplementary Figure 3: comparison of CD4+T cell subsets between the groups before treatment and weeks 6, 12, or 24 after the treatment. (A) and (B) represent the absolute number and percentage of the Th1 cells and (C) and (D) represent those of the Th2 cell levels, respectively. Two-tailed unpaired t-test was used to compare the disease activity measures between the sirolimus and conventional groups. #p < 0.05 relative to the baseline (week 0) in the sirolimus group (red); ∗p < 0.05 compared between the groups. [file 7684352.f1.docx]

| The random number of patients | Group | Age (years) | Sex | ESR changes pre and post therapy | DAS28 changes pre and post therapy | Prednisone dose (mg per day) | Other medications | Description of sirolimus related adverse effects |
| --- | --- | --- | --- | --- | --- | --- | --- | --- |
| *001 | S | 63 | female | 120-101=19 | 5.30-4.07=1.23 | 10 | meloxicam | None |
| *005 | S | 44 | female | 13-5=8 | 4.21-3.64=0.57 | 5 | leflunomide，Hydroxychloroquine，meloxicam | None |
| *007 | C | 61 | female | 40-18=22 | 5.65-2.63=3.02 | 10 | diprospan，biological agents，Hydroxychloroquine，meloxicam | None |
| *008 | S | 64 | female | 56-43=13 | 4.67-2.77=1.90 | 10 | leflunomide，Hydroxychloroquine，meloxicam | None |
| *009 | C | 59 | male | 80-30=50 | 4.07-3.38=0.69 | 15 | leflunomide，meloxicam | None |
| *011 | S | 52 | female | 55-73=-18 | 4.65-4.85=-0.20 | NA | meloxicam | None |
| *012 | S | 57 | female | 24-26=-2 | 3.23-2.49=0.74 | 10 | loxoprofen sodium,leflunomide | None |
| *013 | C | 37 | female | 35-70=-35 | 4.05-4.26=-0.21 | 10 | NA | None |
| *014 | S | 49 | female | 31-14=17 | 4.26-3.24=1.02 | NA | diprospan,biological agents，leflunomide，celecoxib | None |
| *015 | S | 52 | female | 35-16=19 | 4.72-3.12=1.60 | NA | leflunomide，total glucosides of paeony capsules,loxoprofen sodium | None |
| *016 | S | 56 | female | 33-22=11 | 4.19-3.56=0.63 | 15 | meloxicam | None |
| 017 | C | 37 | female | 17.8- | 4.27- | NA | methotrexate，celecoxib | None |
| *018 | S | 54 | male | 57-26=31 | 4.23-3.20=1.03 | 5 | leflunomide，levofloxacin | None |
| *019 | S | 63 | male | 36-25=11 | 4.55-2.39=2.16 | NA | leflunomide，Hydroxychloroquine，loxoprofen sodium | None |
| *020 | S | 62 | male | 37-26=11 | 5.95-5.64=0.31 | 5 | diprospan，methotrexate，calcium and vitamin D | None |
| *021 | C | 54 | female | 10-14=-4 | 3.76-2.62=1.14 | NA | methotrexate | None |
| *022 | S | 63 | female | 15-18=-3 | 5.12-3.96=1.16 | NA | leflunomide | None |
| 025 | S | 32 | female | 6- | 1.86- | 10 | methotrexate，loxoprofen sodium | None |
| *026 | C | 62 | female | 38-9=29 | 4.22-1.75=2.47 | 3 | leflunomide，etoricoxib，iguratimod | None |
| 028 | S | 54 | female | 88- | 6.89- | 10 | diprospan，biological agents，levofloxacin ，thalidomide | Edema, relief after drug withdrawal for a week |
| *029 | C | 59 | female | 18-24=-6 | 3.63-2.43=1.20 | NA | diprospan，biological agents，leflunomide，meloxicam | None |
| 030 | S | 59 | female | 80- | 6.5- | NA | leflunomide，meloxicam | None |
| 034 | S | 58 | female | 31- | 4.41- | 15 | leflunomide，loxoprofen sodium | None |
| *035 | S | 28 | female | 18-10=8 | 3.88-3.07=0.81 | 10 | leflunomide，meloxicam | None |
| *036 | S | 52 | female | 20-21=-1 | 3.84-2.27=1.57 | NA | leflunomide，Hydroxychloroquine，imrecoxib，iguratimod | None |
| *091 | C | 64 | female | 21-21=0 | 4.79-3.34=1.45 | 13 | leflunomide，celecoxib | None |
| *092 | S | 60 | male | 25-36=-11 | 3.82-2.58=1.24 | 8 | leflunomide，meloxicam | None |
| *096 | C | 47 | female | 34-26=8 | 4.93-4.23=0.70 | 15 | methotrexate，kunxian capsule，etoricoxib | None |
| *098 | S | 48 | female | 24-7=17 | 4.16-1.43=2.73 | NA | leflunomide | None |
| *099 | S | 28 | male | 44-40=4 | 4.19-3.07=1.12 | NA | leflunomide，levofloxacin，iguratimod，diacerein，leuproramine | None |
| *103 | C | 47 | female | 22-12=10 | 3.21-1.60=1.61 | NA | leflunomide，Hydroxychloroquine，iguratimod | None |
| *107 | S | 48 | male | 22-12=10 | 3.96-2.79=1.17 | 10 | loxoprofen sodium,leflunomide | None |
| *108 | S | 42 | female | 48-32=16 | 3.40-3.50=-0.10 | NA | leflunomide | None |
| *112 | C | 40 | female | 32-14=18 | 3.22-1.85=1.37 | NA | Hydroxychloroquine，celecoxib，thalidomide | None |
| *113 | C | 63 | male | 32-20=12 | 3.89-2.87=1.02 | NA | diprospan，biological agents，methotrexate，total glucosides of paeony capsules，etoricoxib | None |
| *117 | S | 58 | female | 120-27=93 | 5.80-2.31=3.49 | NA | meloxicam | None |
| *118 | C | 54 | male | 3-4=-1 | 3.67-1.18=2.49 | 10 | meloxicam，leflunomide | None |
| *119 | C | 53 | female | 28-17=11 | 4.65-3.59=1.06 | 8 | leflunomide，loxoprofen sodium，diacerein | None |
| *121 | S | 56 | female | 57-41=16 | 3.56-3.30=0.26 | NA | biological agents，kunxian capsule，meloxicam | None |
| *123 | S | 47 | female | 24-11=13 | 4.03-2.94=1.09 | 10 | tripterygium wilfordii，meloxicam | None |
| *126 | S | 55 | female | 76-47=29 | 5.12-3.51=1.61 | 5 | leflunomide，diclofenac sodium | None |
| *129 | S | 55 | female | 42-13=29 | 4.95-2.80=2.15 | 20 | Hydroxychloroquine，kunxian capsule，meloxicam | None |
| *130 | S | 59 | male | 5-5=0 | 3.84-1.83=2.01 | 4 | leflunomide，meloxicam | None |
| *132 | C | 47 | female | 45-33=12 | 4.97-4.45=0.52 | NA | biological agents，Hydroxychloroquine，meloxicam | None |
| *133 | C | 62 | female | 43-20=23 | 4.24-4.05=0.19 | 10 | diprospan，biological agents，leflunomide，etoricoxib，kunxian capsule | None |
| *139 | S | 57 | female | 90-62=28 | 4.83-4.05=0.78 | NA | leflunomide，Hydroxychloroquine | None |
| *140 | S | 30 | female | 20-32=-12 | 3.71-4.00=-0.29 | 5 | Hydroxychloroquine | None |
| *142 | S | 39 | female | 17-6=11 | 4.35-3.10=1.25 | 5 | leflunomide，meloxicam，iguratimod | None |
| *144 | C | 54 | female | 16-8=8 | 3.91-1.53=2.38 | 10 | leflunomide，loxoprofen sodium | None |
| *146 | S | 42 | female | 80-44=36 | 8.56-2.99=5.57 | 15 | meloxicam | None |
| *147 | C | 49 | female | 80-65=15 | 4.51-4.25=0.26 | NA | leflunomide，iguratimod，etoricoxib | None |
| *148 | S | 56 | female | 84-66=18 | 5.11-4.40=0.71 | 15 | Hydroxychloroquine，meloxicam | None |
| *149 | S | 45 | female | 62-15=47 | 5.14-2.67=2.47 | 10 | diprospan，biological agents，levofloxacin | None |
| *151 | S | 49 | male | 4-8=-4 | 4.02-3.74=0.28 | NA | leflunomide，Hydroxychloroquine，meloxicamvitamin A | None |
| *153 | S | 55 | female | 14-9=5 | 3.80-2.45=1.35 | 5 | methotrexate，celecoxib | None |
| *155 | S | 53 | female | 75-42=33 | 4.98-5.10=-0.12 | 10 | ibuprofen，diacerein | None |
| *224 | S | 62 | female | 14-8=6 | 4.30-3.15=1.15 | 5 | leflunomide，loxoprofen sodium | None |
| *225 | S | 24 | female | 120-40=80 | 6.32-2.72=3.60 | 10 | leflunomide，loxoprofen sodium | None |
| *239 | C | 44 | female | 9-15=-6 | 3.96-4.04=-0.08 | 10 | biological agents，leflunomide，celecoxib，leuproramine，diacerein | None |
| *240 | S | 36 | female | 5-19=-14 | 3.78-5.83=-2.05 | 10 | lornoxicam | None |
| 244 | S | 47 | female | 15- | 4.36- | NA | Hydroxychloroquine，celecoxib | None |
| 246 | C | 44 | female | 16.4- | 3.24- | 20 | methotrexate，loxoprofen sodium | None |
| S=Sirolimus Group C=Control Group DAS28=Rheumatoid Arthritis Disease Activity Index ESR=Erythrocyte Sedimentation Rate NA=Not Applicable *Patients who completed the study | | | | | | | | |

**Table S1:** Clinical details of patients enrolled in the study.


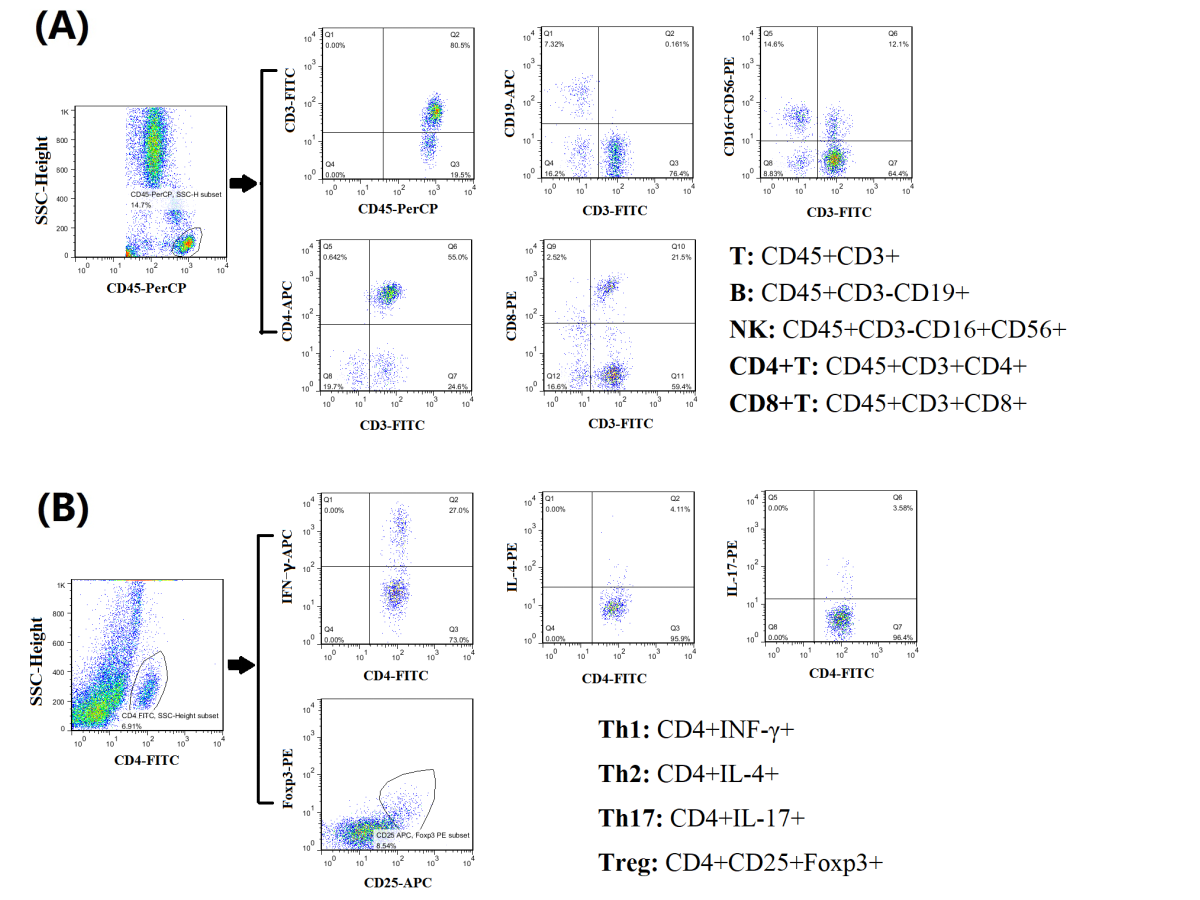


**Figure S1:**  Phenotypic characterization of lymphocyte subpopulations by Flow cytometry. (A) Representative flow cytometry analysis of peripheral lymphocytes.T: CD45+CD3+; B: CD45+CD3-CD19+; NK: CD45+CD3-CD16+CD56+ NK; CD4^+^T: CD45+CD3+CD4+; CD8^+^T: CD45+CD3+CD8+. (B) Representative flow cytometry analysis of CD4+ T cell subsets. All dot plot analysis is of CD4+ gated lymphocyte. Th1: CD4+INF-γ+; Th2: CD4+IL-4+; Th17: CD4+IL-17+; Treg: CD4+CD25+Foxp3+.


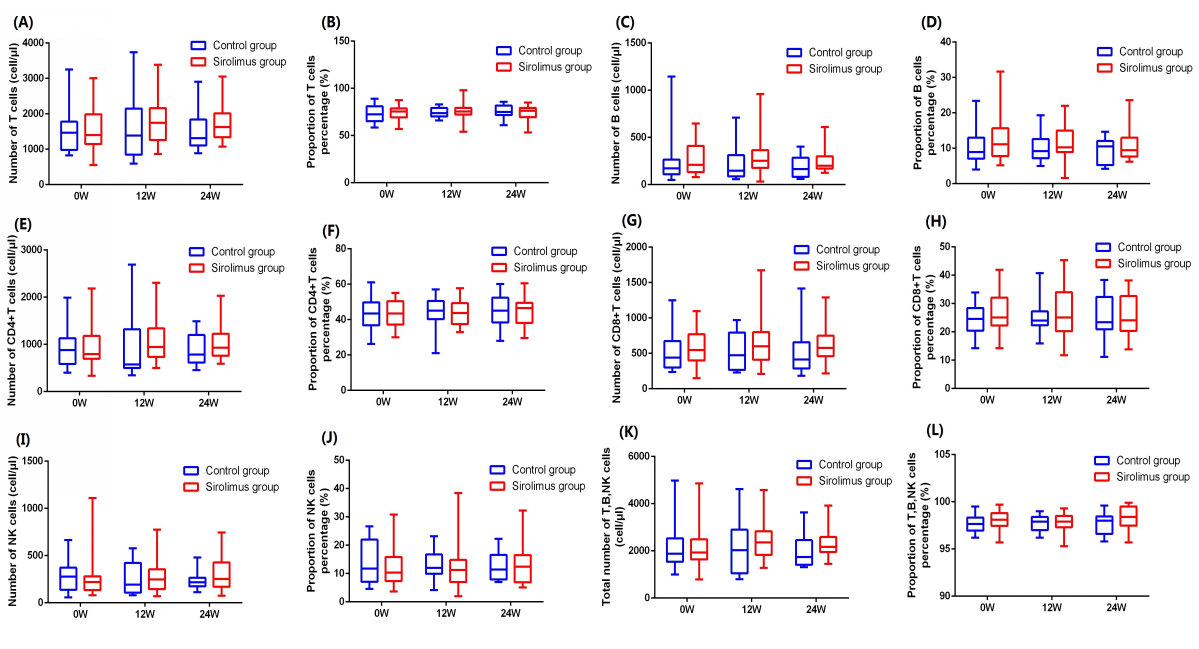


**Figure S2:**  Comparison of lymphocyte subpopulations between the groups at visit 1,4 and 5. (A, B) represent T cell level, (C, D) B cell level, (E, F) CD4+T cells level, (G, H) CD8+T cells level, and (K, L) total lymphocyte cells level. Effects of treatments were assessed by repeated measures analysis using a mixed-effects model. Two-tailed unpaired *t*-test was used to compare the disease activity measures between sirolimus and conventional groups. No significant differences observed at each visit and between the groups.


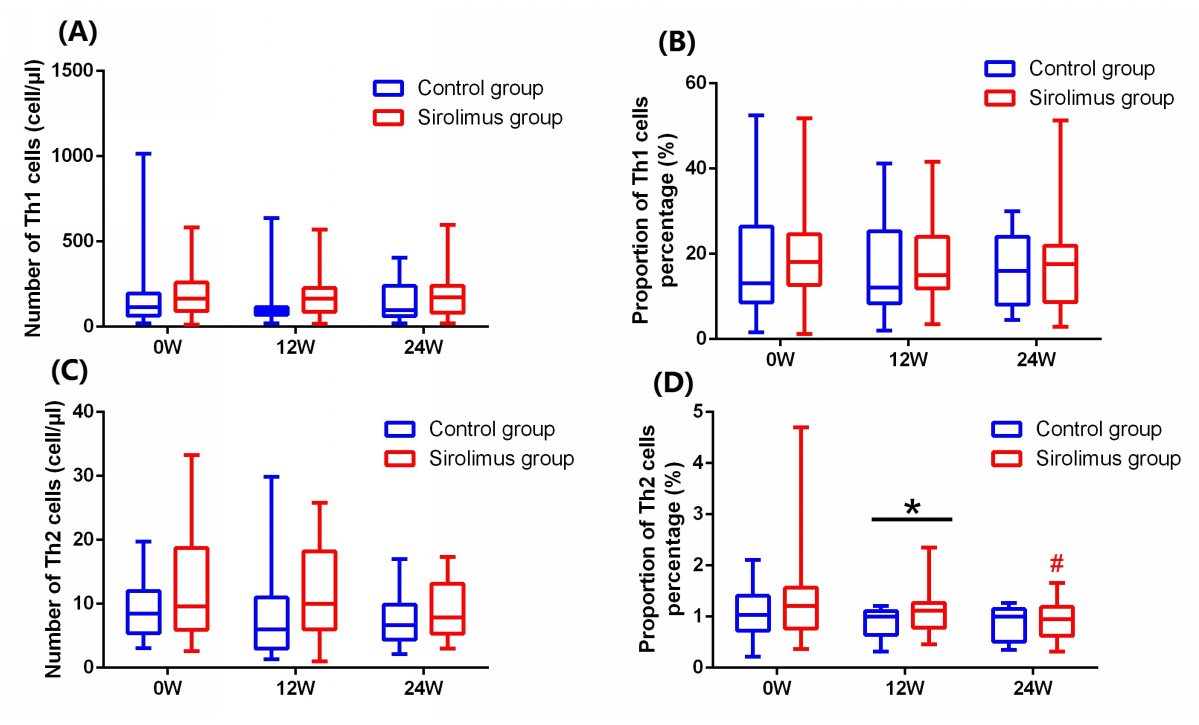


**Figure S3:**  Comparison of CD4+T cell subsets between the groups before treatment and week 6, 12 or 24 after the treatment. (A, B) represent the absolute number and percentage of Th1 cells and (C, D) represent Th2 cell levels, respectively. Two-tailed unpaired *t*-test was used to compare the disease activity measures between sirolimus and conventional groups. #*p*< 0.05relative to baseline (week 0) in sirolimus group (red).; * *p*< 0.05 compared between group.
